# Large naturally-produced electric currents and voltage traverse damaged mammalian spinal cord

**DOI:** 10.1186/1754-1611-2-17

**Published:** 2008-12-30

**Authors:** Mahvash Zuberi, Peishan Liu-Snyder, Aeraj ul Haque, David M Porterfield, Richard B Borgens

**Affiliations:** 1 Department of Agricultural and Biological Engineering, Purdue University, West Lafayette, IN, USA; 2 Department of Biomedical Engineering, Brown University, Providence, RI, USA; 3 Department of Agricultural and Biological Engineering, Purdue University, West Lafayette, IN, USA; 4 Department of Agricultural and Biological Engineering, Department Horticulture and Landscape Architecture, Weldon School of Biomedical Engineering, Bindley Bioscience Center, Purdue University, West Lafayette, IN, USA; 5 Center for Paralysis Research, School of Veterinary Medicine; Weldon School of Biomedical Engineering, College of Engineering; 408 S. University St., Purdue University, West Lafayette, IN, USA

## Abstract

**Background:**

Immediately after damage to the nervous system, a cascade of physical, physiological, and anatomical events lead to the collapse of neuronal function and often death. This progression of injury processes is called "secondary injury." In the spinal cord and brain, this loss in function and anatomy is largely irreversible, except at the earliest stages. We investigated the most ignored and earliest component of secondary injury. Large bioelectric currents immediately enter damaged cells and tissues of guinea pig spinal cords. The driving force behind these currents is the potential difference of adjacent intact cell membranes. For perhaps days, it is the biophysical events caused by trauma that predominate in the early biology of neurotrauma.

**Results:**

An enormous (≤ mA/cm^2^) bioelectric current transverses the site of injury to the mammalian spinal cord. This endogenous current declines with time and with distance from the local site of injury but eventually maintains a much lower but stable value (< 50 μA/cm^2^).

The calcium component of this net current, about 2.0 pmoles/cm^2^/sec entering the site of damage for a minimum of an hour, is significant. Curiously, injury currents entering the ventral portion of the spinal cord may be as high as 10 fold greater than those entering the dorsal surface, and there is little difference in the magnitude of currents associated with crush injuries compared to cord transection. Physiological measurements were performed with non-invasive sensors: one and two-dimensional extracellular vibrating electrodes in real time. The calcium measurement was performed with a self-referencing calcium selective electrode.

**Conclusion:**

The enormous bioelectric current, carried in part by free calcium, is the major initiator of secondary injury processes and causes significant damage after breach of the membranes of vulnerable cells adjacent to the injury site. The large intra-cellular voltages, polarized along the length of axons in particular, are believed to be associated with zones of organelle death, distortion, and asymmetry observed in acutely injured nerve fibers. These data enlarge our understanding of secondary mechanisms and provide new ways to consider interfering with this catabolic and progressive loss of tissue.

## Background

It is the early events following severe injury to the brain and spinal cord that have received significant attention. In part this is to better understand the progression of tissue damage and to develop means to interfere with it. Many factors have been considered to play a role in the collapse of the spinal cord and brain architecture within hours to days post-injury. This period of time, variable in extent, is usually referred to as "secondary injury" [1], the primary injury being the acute mechanical insult to the tissue. The biology/pathology forming the basis for secondary injury in the mammalian CNS includes – but is not limited to – particular biochemistries such as: the formation of reactive oxygen species (so-called free radicals) and the initiation of lipid peroxidation of the inner membrane which begins immediately after mechanical damage to CNS cells [2,3] the formation of endogenous toxins that accumulate within damaged neurons and their processes [3]; the loss of myelin and the associated collapse of electrophysiological conduction [1,4]; and the initiation of both apopotosis and progressive necrosis by chemically-mediated events. These are the two main forms of cell death in adult animals, and each plays a role in the demise of CNS parenchyma after mechanical damage [3,5].

The role of the endogenous bioelectric (ionic) current and the generation of steady DC voltages within damaged CNS parenchyma have been largely ignored. The usual review and narrative of the progression of secondary injury mechanisms begins with the mention of calcium entry into the cytoplasm of cells and their processes (in the special case of glia and neurons). Likewise it is left to the reader to surmise what mechanisms lead to the influx of Ca^2+ ^[6]. In fact it is assumed that the majority of ions in the extracellular milieu – Na^+ ^and Ca^2+ ^which enter the cell, and K^+ ^which leaves it – are a simple matter of diffusion down their concentration gradients. Of course this is an electrochemical gradient, but there are other forces at work that drive the initial biophysical and electrophysiological process which initiate and prolong the progression of secondary injury.

Here we present the first non-invasive measurements of very large (≤ mA/cm^2^) electric currents driven into the site of damage in fully adult mammalian (guinea pig) spinal cords. This is a true DC electrical current, carried by ions, that is driven by the Electromotive Force (EMF) of the surrounding intact cell membranes. While this electrical injury (both current and voltage mediated) cannot be separated from the earliest pathophysiology associated with mechanical injury, neither can they be ignored. We discuss the role such huge levels of electric current may play in the responses of nerve cells to damage, in particular, the ionic components of this ionic/bioelectric current and possible means to interfere with it.

## Results

### Peak currents and their dynamics

**Figure 1:**
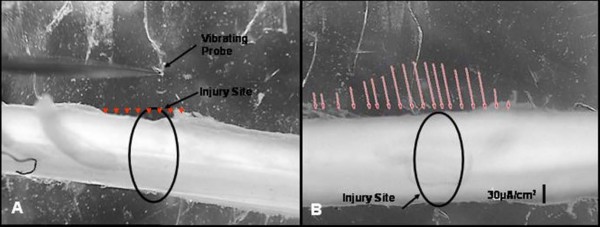
**Computer monitor captures of raw data of 2 D scans of the guinea pig spinal cord**. A is prior to Injury, and B is the same cord after crush of the tissue with a laboratory fabricated forceps possessing a détente. Vectors (as arrows) reveal inwardly directed current entering at peak magnitude at the locus of the crush injury and declining in magnitude with distance from this site. Measurements were made approximately 15 mins post injury. The current was balanced by extracellular current measured entering the tissue in relatively undamaged regions of white matter bordering the injury zone. Pre-injury current along uninjured spinal cords was no different than background (arrowheads in A). The background current was offset by taking a reference measurement before actual injury currents were measured.

**Figure 2:**
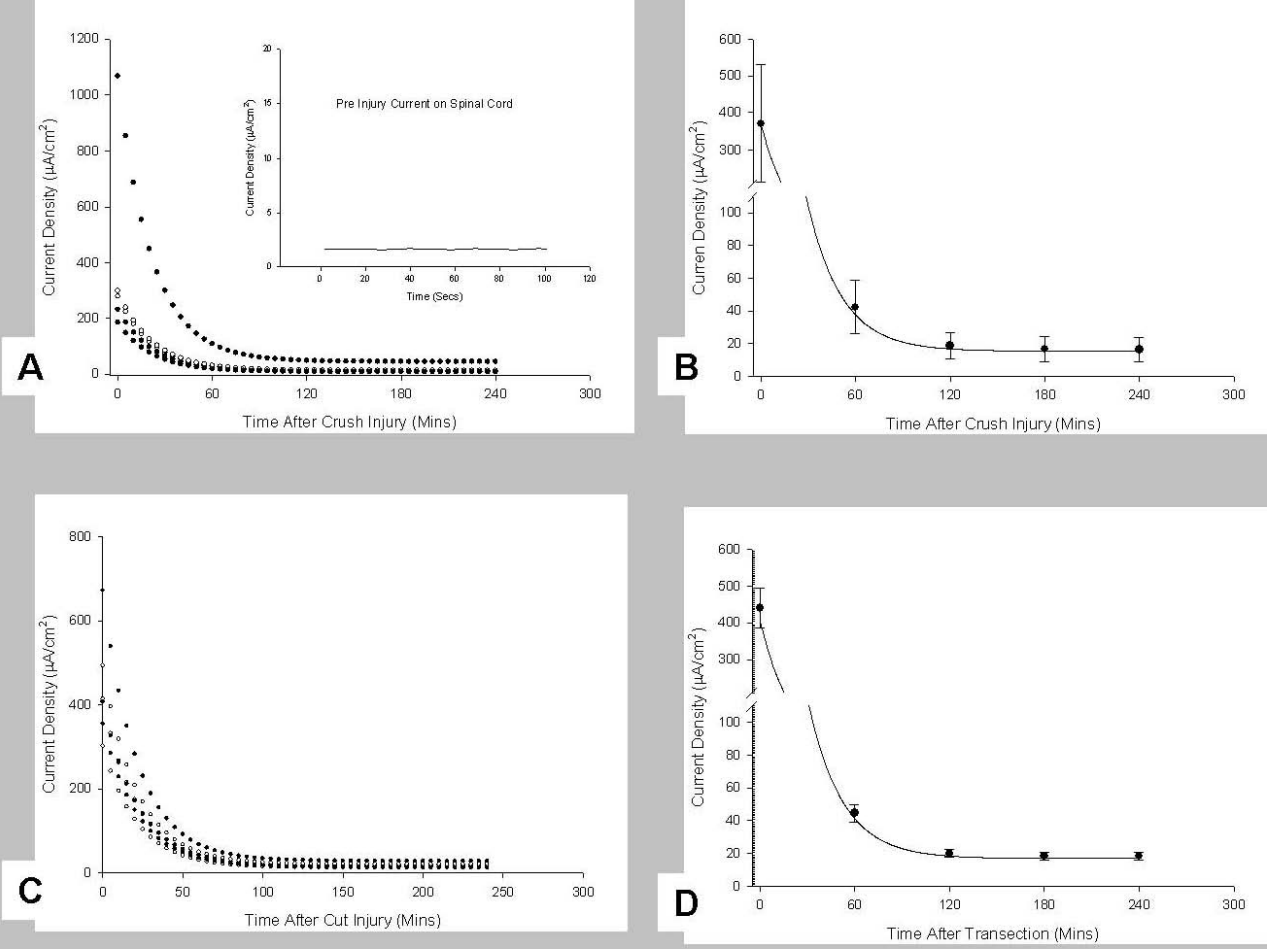
**Decline in peak current density with time after injury**. A shows scatter plots of raw data on 5 individual spinal cords for the first 4 hours after a crush injury, while B shows the Mean and SEM of these same data. Note that one cord produced over 1 mA/cm^2 ^of electric (ionic) current entering the lesion. C and D are similar plots, but derived from measurements made on transected spinal cords. Note the initial current densities are similar, but transected spinal cords appear to have a higher magnitude of stable current entering the transection site relative to crush injuries. All measurements were made 50 μm from the injury sites. These raw data were then corrected using a modified three parameter exponential decay model to determine the injury current densities at the surface of the cord. Pre-injury currents along all of these spinal cords were the same as background (See Fig 1). The background current was offset by taking a reference measurement before actual injury currents were begun. Inset in A shows a representative pre-injury current measurement made on the spinal cord in A. All of these measurements were made on ventral regions of the spinal cord.

Fig. 1A and 1B show the spinal cord in the recording chamber, before and after a focal crush injury. Note the lack of significant ionic current traversing the cord prior to injury and significant inwardly directed current entering the crush site, which declines with distance from the epicenter. Fig. 1B shows that the fall in current density with distance from the epicenter of the injury site is approximately linear relative to the exponential decline with time after injury (Fig 2). Fig. 2A and 2B show the *peak *current densities entering a crush site on 5 spinal cords and their decline with time. Fig. 2A is a scatter plot of data revealing outlying points, while B presents the means and SEM of the same 5 samples. Note that initial densities are approximately 0.2 – 0.3 mA/cm^2^, with one sample driving more than 1 mA/cm^2 ^into the injury site. This was not uncommon as currents of this magnitude were also observed in pilot experiments. The more stable current entering the injury site from about 1 – 4 hours of measurement post-crush was on the order of 40 μA/cm^2 ^– representing more than 10 fold decay in the magnitude of the current within the first few minutes of the injury. Fig. 2C and 2D are identical plots yet made from data taken from transection injuries where the cord was cut with iridectomy scissors. This was a complete transection of the spinal cord, and measurements were made with one of the vibrating probes axis perpendicular to the cut face, i.e. normal to the cut face. Severing white matter appears to produce similar densities of initial current, and the decline is not significantly different than that observed after crush injuries. The number of samples scanned in these initial measurements does not permit a strong statement concerning this apparent difference between "crush or cut" injuries; at first look, however, they do not seem substantially different.

### Modeling instantaneous injury currents and the definition of dorsal and ventral differences in them

One potentially important observation was the apparent dominance of injury currents entering the ventral surface of the spinal cord relative to those measured from the dorsal surface. To more keenly understand and verify these observations required an improvement in our extrapolation of current densities at the surface of the cord, at the instant of injury, and at times after that. This was necessitated by the desire to compare these different regions of the injury in the spinal cord.

**Figure 3:**
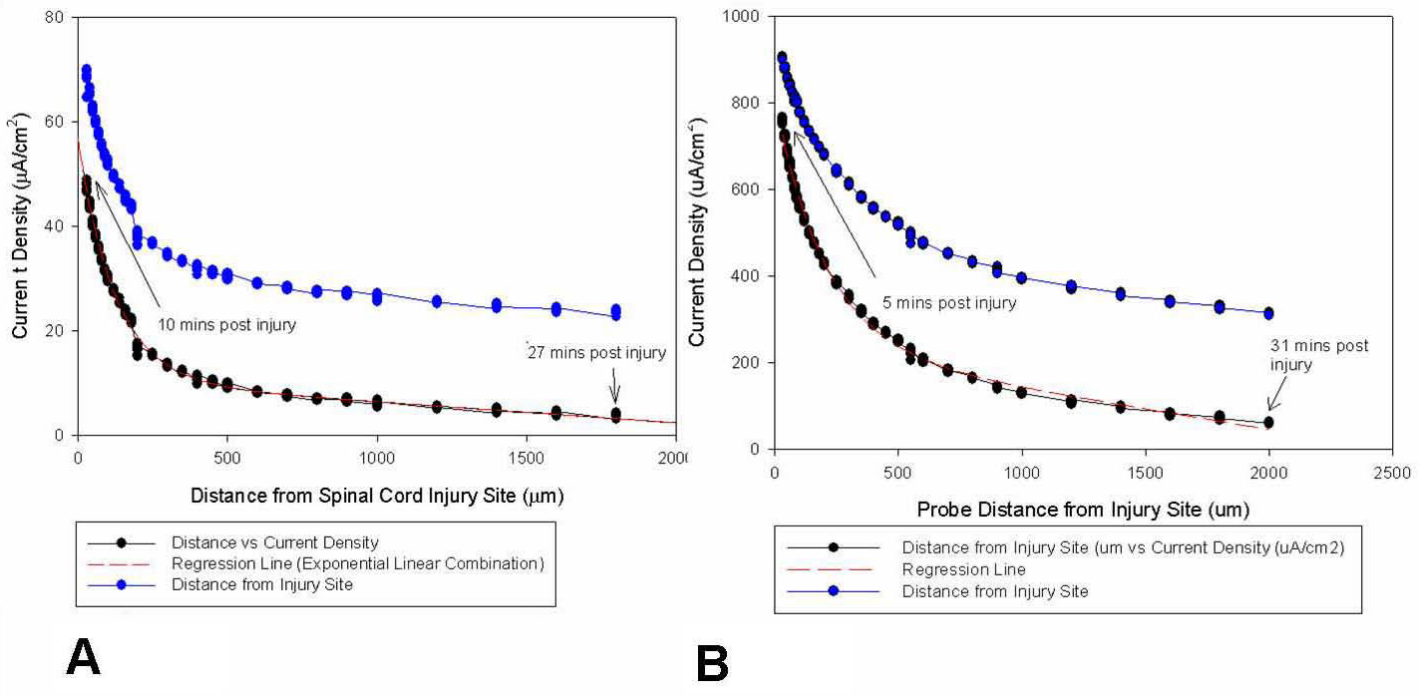
**Dorsal versus ventral injury current**. Data obtained from "Step-back" experiments on two guinea pig spinal cord crush injury sites. A shows recordings made near the dorsal portion of spinal cord. The first measurement was taken 10 minutes after creating the crush injury at a distance of 30 μm from the injury site. B shows similar recordings obtained near the ventral portion of a spinal cord. The first measurement was recorded also approximately 30 μm from the injury site, and 5 mins after the injury was inflicted. Note the differences in scale of magnitude. The vibrating probe was then stepped back at smaller distances at first and then larger distances afterwards, until the current decayed to a steady level. As explained in the text, an "exponential linear combination" model was used for curve fitting and has been extended to show the theoretical current value at the injured spinal cord surface (x = 0). This model is different from the artificial source model because of obvious differences in the current source geometry (point source as compared to the complicated geometry of spinal cord injury site). The step-back data was corrected for current decay due to time using the time adjustment model and is plotted as well (blue dots). Note that now the current density at distance of up to 2 mm from injury site is much higher. If this plot were to be extended, it would touch the x-axis "cms" away from the injury site. This implies that the injury current field is much larger than expected and might explain the extent to which a focal injury can propagate in distance and time.

Current Densities from 5 separate cords, showing *peak current density *measurements from the dorsal and ventral surface of the injury zone are given in Table 1. The crush injury currents produced in the ventral portion of spinal cords had a median value of 278.8 μA/cm^2^, a minimum value of 184.7 μA/cm^2 ^and a maximum value of approx. 1.06 mA/cm^2^. In contrast, the crush injury currents entering the dorsal portion of the spinal cords were almost 3 times lower, with a median value of 60.3 μA/cm^2^, minimum value of 32.2 μA/cm^2 ^and a maximum value of 72.3 μA/cm^2 ^(see Table 1). Having expected this difference (see Discussion), paired, one-tailed evaluation of the data presented in Table 1 reveals that these data are statistically significantly different (P = 0.004), while a non-parametric two-tailed evaluation (that holds no assumptions on the distribution of the data) still reveals the data to be extremely significantly different (P = 0.008). Fig 3A and 3B provide graphical representation of dorsal and ventral current densities. This initial evaluation shows that the magnitude of endogenous current entering a crush injury to the ventral portion of the spinal cord (B) may be as much as 10 times larger than that produced by an injury to the dorsal portion (A). Since the step-back experiments take a finite amount of time to perform, the temporal current loss needs to be accounted for. Figs. 3A and 3B were adjusted by using a temporal current density decay correction model, which is also plotted (blue). Note that the current density at a distance of up to 2 mm from injury site is much higher. If this plot were to be extended, it would touch the x-axis "cms" away from the injury site. This implies that the injury current field is much larger than expected and helps explain the extent to which a focal injury can propagate.

**Table 1:** Peak spinal cord injury currents measured on ventral and dorsal portion of guinea pig spinal cords.

**n**	**Vertical portion of spinal cord (μA/cm^2^)**	**Dorsal portion of spinal cord (μA/cm^2^)**
1	231.61	69.03

2	278.79	32.23

3	298.53	44.93

4	184.75	60.29

5	1065.79	72.32

Five samples each of the ventral and dorsal portions were used. The crush injury currents produced in the ventral portion of the spinal cords had a median value of 278.79 μA/cm^2^, a minimum value of 184.75 μA/cm^2^and a maximum value of almost 1.06 mA/cm^2^. Alternately, the crush injury currents produced on the dorsal portion of the spinal cords are almost 3 times lower, with a median value of 60.29 μA/cm^2^, minimum value of 32.23 μA/cm^2 ^and a maximum value of 72.32 μA/cm^2^. Comparison of these data using a two-tailed Mann-Whitney non-parametric test revealed the differences between dorsal and ventral ionic current flow to be extremely significant (P = 0.0079).

**Figure 4:**
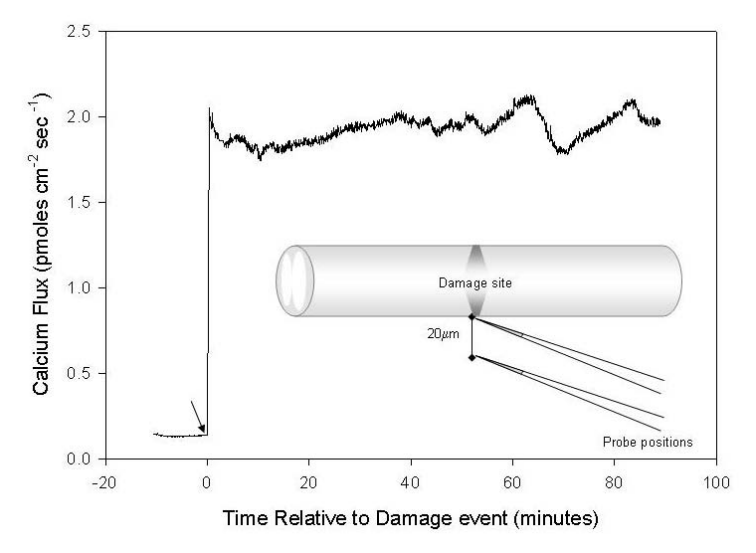
**Calcium entry in damages spinal cords**. The electrical record is the output from a 2 D calcium-specific probe vibrated approximately 20 μm from the surface of the damaged region of crushed spinal cord. Note the stable value of ~2 pmoles/cm^2^/sec over the hour of recording.

When a calcium specific probe was used to measure peak currents (Fig 4), a mean concentration for 0 to 20 minutes post injury was 1.86 +/- 0.12 pmoles/cm^2^/sec; at 20–40 minutes 1.95 +/- 0.10 pmoles/cm^2^/sec; and at 40–60 minutes, 1.93 +/- 0.10 pmoles/cm2/sec.

## Discussion

The "battery" (EMF) driving this flow of current into axons is the inwardly negative potential (~50 – 70 mV) across undamaged cellular membrane at variable distances from the site of mechanical damage. It is convenient to consider that the inwardly negative membrane potential(s) "short-circuit" ionic current through the compromised integrity (hence significantly reduced resistance) of damaged membranes, producing trans-axonal DC current flow (reviewed in [1]).

A rapid decay of initial current density was expected and had been reported in measurements made from complete transection of individual giant reticulospinal axons in the ammocoete lamprey [7]. Even given the striking anatomical differences between this proto-vertebrate model, possessing 40–60 μm diameter unmyelinated axons, and the mammal, the dynamics of current decline were remarkably similar. Initial densities were on the order of less than 1 mA/cm^2 ^entering lamprey giant axons and nearby parenchyma, reaching stable densities in the tens of μA/cm^2^. The ammocoete lamprey's entire brain and spinal cord can be removed and maintained for up to a week in organ culture since the ammocoete's CNS is not intrinsically vascularized. These facts permitted measurements of the injury current entering the transection for many days after injury in the lamprey but cannot be done with the mammalian spinal cord *ex vivo*. Based on the similarities of these data, it is reasonable to expect the plateau current in the mammalian cord is also likely to persist for many days post-injury

### Geometric and tissue considerations

The net current densities reported here are the sum of all internally directed cellular injury currents minus any outwardly directed current that may be produced by the epithelial-like investments of the spinal cord. Epithelial driven ionic currents will be outwardly directed (usually abluminal) while cellular injury currents are driven into damaged cells. It is the *net current*, i.e. the ions that carry the current into cytosol, that is the prime culprit in the process of early secondary injury. It has been reported that both types of injury currents can occur simultaneously and influence the dynamics of the net current flow into cells, for example, the outflow of "stump currents" subsequent to the amputation of salamander limbs [8], and in the case of streaming potentials (injury current) in mammalian bone [9].

The elongate geometry and parallel arrangement of the axons of mammalian white matter supports the dynamics of a strong and steady ionic current flow through and along the long axis of the spinal cord. Injury to axons close to the cell body usually results in death of the cell [10]. This death is related to the amount of calcium influx and the distance that it penetrates along the axon as Ca^2+ ^invades the cytosol. The closer to the cell body that the axon is injured, the greater the chance that the entire cell will succumb to the injury. In white matter long tracts, the site of damage is often very far from the cell bodies giving rise to these fibers, thus the cell and the proximal segment of fiber remain viable until the spontaneous sealing of the axonal membrane. The resistivity of white matter in the long axis of the spinal cord (300 – 400 Ω cm) – is 4–5 fold smaller than the resistivity measured in the transverse direction (1500 – 2500 Ω cm). Thus, mammalian spinal cord white matter is strongly anisotropic in terms of its electrical conductance in the longitudinal and transverse axis. This ensures a preferential circuit along and not across the axons making up the tissue, further suggesting a reduced loss in the peak magnitudes of longitudinal current by tangential current flow in white matter. Furthermore, this anisotropy would be expected to support a standing DC voltage gradient in the long axis of the cord, inside and outside of the axons that make it up. This voltage would be internally positive at the site of the injury and less positive (i.e. more negative) at distances from the point of injury in the tissue of the spinal cord. This is true no matter what types of axons are considered, as the steady DC voltage gradient will be expressed in this polarity independent of the direction of impulse conduction, i.e. in ascending or descending white matter tracts (see below).

### Ionic composition

Ionic current entering the injury site is carried by the ions most concentrated in the external medium [7,11]. This would be Na^+^, Cl^- ^and Ca^2+^. Of particular interest is the Ca^2+ ^component of this current, since the enormous elevation in cytosolic Ca^2+ ^is correlated to the complete collapse of the cytoarchitecture of axons at the point of damage in addition to facilitating, as a necessary co-factor, many catabolic biochemical cascades ending in necrosis and apoptosis. This role in the catabolism/destruction of cells from outside cytosolic invaders has been well understood since the pioneering work of William Schlaepher and Richard Bunge [12].

We have emphasized the role of the calcium ion; however this is only in recognition of the importance given it in this literature. In fact, calcium is only one of several key players in the electrical/ionic disturbance after mechanical injury. Increase in cytosolic Na^+ ^also induces a secondary rise in Ca^2+^, as this triggers Ca^2+ ^release from intracellular stores. This role of Na^+ ^initiated Ca^2+ ^release has also been well described since the seminal studies of Carafoli and Crompton [13] but is usually largely ignored. This elevation in intracellular Ca^2+ ^is thus expressed as a gradient itself: high at the point of damage and falling with distance (hence the potential difference) along the long axis of the axon. Using a calcium specific ion selective electrode, we have measured about 1.9 – 2 pmoles/cm^2^/sec of free calcium entering the site of damage. In such an electrode, a Liquid Ion Exchange (LIE) based microelectrode is the sensor, rather than the platinum black tipped probe. Given that the intracellular concentration of calcium is on the order of nmolar – tenths of μ molar, we suspect again that the lessons learned in fish giant axons may be true here as well: that a calcium gradient in the axoplasm dominates and at saturation levels near the site of injury to cells and tissues. Note our measurements in the mammalian cord do not show much of a decrease in Ca^2+ ^entry at the injury site over the first hour, while the net current falls in magnitude over this time. This influx of Ca^2+ ^is of course associated with the complete destruction of cytoarchitecture but may have more subtle effects. This might also indicate that the diffusion dependent element of the current is composed of primarily an influx of Na^2+^, Cl^-^, Ca^2+ ^and efflux of K^+ ^and is the major contributor to the large initial net injury current. The plateau current, however, indicates the presence of the driving EMF developed by the adjacent undamaged cell membranes which continue to drive this smaller component into the insult. This keeps driving an ionic front of which Ca^2+ ^is a major part even hours after the initial injury along the initially uninjured portions of the spinal cord. This inevitably leads to spread of the injury. This notion is supported by the observation that the Ca^2+ ^current even 60 mins post injury is not much different from that observed immediately post injury.

We believe the fall in potential along the axis of the spinal cord from the injury site associated with the fall in free calcium may likely be correlated to the zones of cytoplasmic and organelle disruption that extend themselves along the long axis of the axon [14,15]. Furthermore, this gradient in free calcium has been visualized in the tips of severed Lamprey giant axons using the florescent probe for Ca^2+ ^(FURA II) [16]. The distal to proximal *fall in the voltage associated with injury currents within the axon *includes zones of peculiar organelle derangements with distance from the disruption, particularly mitochondrial rearrangement. In this zone of damage, mitochondria are arranged in "chains," with their long axis aligned with the length of the axon [14,15]. We believe this may be an expression of voltage mediated effects of ionic current flow through the nerve fiber, acting to impose a preferred orientation on charged components (organelles and pieces of organelles) within the electrical field associated with the intra-axonal responses to the ions (principally Ca^2+ ^and Na+) that carry the current.

Finally the exodus of K^+ ^is singularly competent to shut down the conduction of nerve impulses in locally damaged axons and thus contributes to the total physiological and behavioral deficit observed immediately after acute CNS damage.

### Dorsal vs. ventral

The difference between dorsal and ventral injury currents might have been expected, having its root in the anatomical differences in mammalian white matter of these regions. The dorsal half of ventral white matter, closest to the ventral horns of gray matter, contains mainly small- and medium-diameter axons. In contrast, more ventral regions possess similar caliber spectra – but also comparatively large-diameter axons – approaching or exceeding 10 μm in diameter [17]. While membrane voltages collapse at or very near the actual site of mechanical damage at distances from this region, a significant EMF is maintained across axolemmas by the physiological pumps residing in all normal membranes. Thus a greater surface area of membrane associated with greater numbers of large caliber axons (indeed the "batteries in series") may likely relate to the observed differences between ventral and dorsal white matter. In the same breath, we admit that this answer is hypothetical and may only be a partial explanation among several. For example, one could surmise that the resting potential of dorsal axolemmas might be lower (hence the EMF supporting the injury current) though we do not know of any physiological measurements suggesting this.

### Historical perspectives

The general phenomenology of large magnitude and significant injury current entering spinal cords could be predicted from classical studies of injury potential or demarcation currents from the mid-nineteenth century to the early part of the 20^th ^century by the discoverer of the Action Potential (Emil DuBoise-Reymond; 1818 – 1896) and other 19^th ^century physiologists. These voltages, sampled with galvanonmeters, were extracellularly negative at the site of damage relative to positions farther away and were independent of the conduction pathway. This suggested an axial current flow in nerves produced by injury that appeared to contradict the orthodromic propagation of action potentials in a preferred direction. This quandary was rejected by the influential developmentalist Paul Weiss (1898 – 1989) who strongly condemned "demarcation potentials" as measurement artifacts and also condemned the early observations of preferential direction of growth of neurites in culture to an applied voltage gradient, after failing to duplicate them [18]. As it turned out, Weiss got it completely wrong on both counts. Raphael Lorente de No', the student of Santiago Ramon y Cahal in Madrid, wrote in 1947 "*It appears now that Weiss's explanation was erroneous and therefore the observation of the classical authors were significant, even though they cannot be regarded as a proof that an axial current flows in nerve*." ([5], page 92; he was addressing the normal state of an undamaged nerve). Lorente de No's continued neurophysiological studies brought him to the understanding that the failure of action potentials at a position very near to the end of a severed segment of nerve was related to the decreased polarization of membrane, associated with the "demarcation potential" in his view. He wrote: "*The explanation of the phenomenon must rather be based on the circumstances attending the decrease in the demarcation current that had been produced by the injury*" [5], page 450]... *It is thinkable; therefore, that the continued flow of the demarcation current into the last few millimeters of a regenerating nerve is a mechanism by means of which energy is transferred to the regenerating end from points at some distance from it." *([5], Page 459; he is discussing axonal regeneration). These issues and controversies evaporated with the birth of the microelectrode age a decade later; however, they bear special recognition here.

Finally we note the now well established clinical use of applied DC voltages arranged parallel with the orientation of white matter in severely injured human spinal cords [1,19-21]. One suggested mechanism of action underlying the preservation of anatomy and subsequently behavioral recovery is a reduction in retrograde degeneration in nervous tissue in response to distally negative gradients of applied DC voltage [1,11]. It is unlikely that an artificially imposed voltage could be used as a therapy in the first minutes after an injury as it is now being used in severe acute spinal cord injury days later [21]. However our findings should reinvigorate the possibility of using calcium blockers if they could be safely administered for a very short time at the site of an accident.

## Conclusion

A very large (≤ 1.0 mA) bioelectric current enters the region of damage in the mammalian spinal cord. It is driven by intact "battery" of cell membranes in undamaged adjacent regions. This magnitude of current is similar in both cut and crushed spinal cords. The magnitude falls rapidly by more than an order of magnitude within minutes of the injury. This ionic current is related to the catastrophic destruction of the anatomy of crushed and cut fibers, extending away from the local site of the insult. Particularly important is the calcium component of the current which enters in concentrations of ~2 picmoles of Ca^+2 ^per second per square centimeter. Increases in ionic Ca^+2 ^above its physiological range is related to the destruction of cell architecture and the enabling of catabolic enzymes in the cytosol as an obligatory cofactor. Curiously, levels of current entering the ventral region of the spinal cord were greater than the injury current entering the dorsal regions of crushed spinal cords. Interfering with the Ca^+2 ^mediated destruction dependent on the ionic current of injury in the early acute phase of the injury might be considered as a means of ameliorating the effects of secondary injury.

## Materials and methods

### Isolation of the spinal cord

Guinea pig spinal cords were isolated using previously specified techniques [22-24]. Ketamine (80 mg/kg), xylazine(12 mg/kg), and acepromazine(0.8 mg/kg) were used to anaesthetize adult guinea (350–500 gms). The guinea pig hearts were then perfused with 500 ml of oxygenated Krebs solution [124 mM NaCl, 5 mM KCl, 1.2 mM KH_2_PO_4_, 1.3 mM MgSO_4_, 2 mM CaCl_2_, 20 mM dextrose, 26 mM NaHCO_3 _and 10 mM sodium ascorbate], equilibrated with 95% O_2 _and 5% CO_2 _to remove blood and lower the body temperature. The vertebral column was excised, spinal cords quickly removed and immersed in cold Krebs solution. All animal use received prior approval by the Purdue University Animal Care and Use committee, in strict accordance with Federal, State, and University guidelines.

### Handling and compression of spinal cords

The ~35 – 40 mm long spinal cords were kept at a room temperature until use and the Krebs solution was replaced every 20 minutes. A 60 mm silicone polymer (sylgard) bottomed petri dish was used to mount the spinal cord for electrophysiological recordings. Stainless steel minutien pins (0.1 mm) obtained from Fine Science Tools (Foster City, CA) were used to carefully pin down the spinal cord at its ends. The crush/compression injury was made with a laboratory – fabricated forceps possessing a détente to help standardize the extent of compression between cords [25]. All spinal cord injuries were timed using a stop watch and the time that elapsed between the injury and the actual recording of the data was subsequently recorded. A constant perfusion was provided in the petri dish to ensure a continuous supply of fresh Krebs solution to the spinal cord while the experiments were being performed.

### Vibrating electrodes for the measurement of extracellular current

Measurements were made with non- invasive one dimensional (1 D) and neutating (or 2 D) probes for the measurement of extracellular current [7,26,27]. The former gives the density of electric current entering or leaving a biological source normal to its surface with time, while the latter provides this as well as two-dimensional information in the form of current density vectors. Spatial resolution is on the order of 20 μm, and, depending on the resistivity of the bathing media, such probes can detect current densities on the order of picoA/cm^2 ^– far below the resolution required here. Current Vectors are displayed as raw data by software and are superimposed over the digital video image and captured by digital image acquisition.

Microelectrodes used for fabricating the vibrating probes were Pt/Ir electrodes (Micro Probe Inc, Gaithersburg, MD) with a 3 – 5 μm exposed tip, while the rest of the electrode was insulated. The tip of the probe was platinum blackened by electroplating to form a 25–30 μm diameter Pt ball. Alternately, the platinum tip can be replaced with one of a calcium specific resin, which then measures only the calcium component of the net current flow. The completed probes were then calibrated in Krebs solution at 37 degrees C as were physiological measurements. A KCl filled glass microelectrode was used as a point source for calibrations (see below). The point source was made using a 1.5 mm internal diameter borosilicate glass capillary tube pulled to a tip diameter of 8–10 μm. This was performed on a David Kopf Vertical puller (David Kopf Instruments, Tujunga, CA). The probe was vibrated at a distance of one tip diameter between its two extreme positions with X and Y frequencies ranging from 250–300 Hz. The probe actually measures the small voltage difference between its extreme positions with a phase/frequency lockin amplifier. This voltage difference together with the known resistivity of the media is used to calculate the bulk current or the current density associated with the sample of interest. The direction of the current vectors shows whether the current is an influx or efflux.

### Calibration

**Figure 5:**
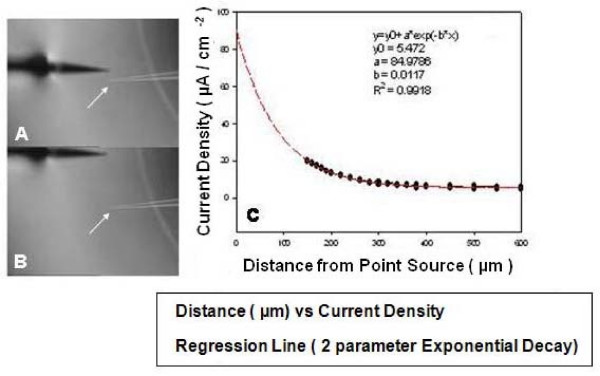
**Probe calibration**. In A, an image of the vibrating probe recording current delivered from a source pipette at a distance of 150 μm is shown. Below it, the vibrating probe has been moved away from the point source to a distance of 600 μm, within the gradient of current delivered by the source pipette. In B, a plot of current density vs distance recorded from such a source pipette (calibration current = 60 nA) recorded by the vibrating electrode. Note that the current density measured 150 μm from the source pipette was approximately 21 μA/cm^2^, a value calculated from this distance and total calibration current (see text). This demonstrates that the vibrating electrode was correctly calibrated.

The 2-D vibrating probe was calibrated using a borosilicate source pipette pulled to a diameter of 8–10 μm. A 60 nA current was passed through the calibration micropipette. In order to null any system offset, the vibrating probe recorded a reference offset in the absence of any applied current before calibrating. The 60 nA source current was then turned on and the probe was vibrated at a distance of 150 μm from the tip of the source pipette, first in the X-axis and then in the Y-axis. If the measured currents at these positions were validated against that expected, the system was considered calibrated. The theoretical current, given the resistivity of the media, at a distance of 150 μm from the 60 nA point source current would be 21.5 μA/cm^2 ^(refer to the legend to Fig 5). Figure 5 shows a representative current density profile measured by a calibrated 2-D vibrating probe near the calibration point source. The recordings were begun 150 μm from the tip of the point source and then the vibrating electrode was backed away from the point source in a stepwise fashion to determine the factor for "fall off" with distance from the point source as a further calibration procedure.

### Temporal and spatial profiles of spinal cord injury currents

Using the vibrating voltage probe to study spinal cord injury we measured a large inwardly-directed injury current at the lesion soon after injury to the spinal cord. This current then decreased rapidly in magnitude to approximately 20% of its original magnitude within 30 minutes (refer to Results above). This decay can be approximated by a 3-parameter exponential decay model:

*y*(*t*) = *y*0 + *a *exp(-*bt*)

where y(t) = current density drop as a function of time, t = time, and y0, a and b are empirically derived, normalized constants.

This expression is the basis of the model for all time-dependent current density measurements.

In a separate study, the vibrating probe was brought to a starting position 50 μm away from the surface of the injury site of the spinal cord. The vibrating electrode was then sequentially stepped back away from the injury site at fixed intervals with the injury current density measured at each point. This step-back, or fall-off, profile provides a reasonable assessment of the spatial profiles of the external electrical field associated with the injury to the spinal cord. This would not be expected to be the same as that data taken from a point source, given the complex and extended geometry of the tissue surface.

An exponential linear combination current decay model provided the best fit for these step-back experiments. We used the formula:

*y*(*x*) = *y*0 + *c *exp(-*dx*) + *ex*

where y(x) = current density drop as a function of distance from injury site, x = distance from injury site, and y0, c, d and e are empirically derived normalized constants. This formula does not account for the current decay with respect to time. To correct for this we applied a correction factor which compensates for this loss to yield the following model;

*y*(*x*) + Δ*y *= *y*0 + *c *exp(-*dx*) + *ex *+ |-*ab *exp(-*bt*)Δ*t*|

Finally, it is of interest to know the magnitude of the current entering the spinal cord at the "instant" of injury (time = 0), and to more properly account for the increased magnitude of the current at the surface of the cord from that actually recorded at the standard measurement position. Given the rapid decline in current with time after the acute injury, and the fact that the probe can not be vibrated any closer than 30–50 μm from the cord's surface without damage, this required some separate study and quantitative normalization of the recorded data.

Calculation of the correction for the current "fall off" due to both time and distance is calculated based on the combination of the formulas presented above in Methods, and is as follows:

Δ*Y*(*x*,*t*) = |[-*CD *exp(-*Dx*) + *E*]Δ*x*| + |-*AB *exp(-*Bt*)Δ*t*|

This current when added to the original current (Y) reveals the injury current at the surface instantaneously after injury for any one experiment:

*Y*(*x*,*t*) = *Y *+ Δ*Y*(*x*,*t*) and,

*Y*(*x*,*t*) = *Y *+ |[-*CD *exp(-*Dx*) + *E*]Δ*x*| + |-*AB *exp(-*Bt*)Δ*t*|

We calculated all of the empirical coefficients using normalized data sets compromising approximately 10 different scans of spinal cord injury current profiles. By using normalized data for this analysis, it was possible to calculate "universal" coefficients. This is necessary since the calculations of constants derived from the raw data can be misleading given the large variations observed when measuring endogenous injury currents, whereas normalized coefficients had less than 1% variability. These coefficients can be used to adjust normalized data sets that then need to be converted back to discrete data.

$$Y(x,t)=Y+\left[\begin{array}{l} \left|-(78.818)(0.0065)\exp(-0.0065x)+(-0.0098)]\Delta x\right|+\\ \left|-(95.83)(0.04625)\exp(-0.04625t)\Delta t\right| \end{array}\right]$$

This derived current density data should not be considered to provide a perfectly accurate picture of the dynamics of injury current flow in the spinal cord; however, it provides the most accurate data extant for understanding the *immediate *decay in physiological currents at any distance from the injury site. Moreover, this method permitted us to both calculate, then compare, the injury current at t = 0 and x = 0 for raw data measured from both ventral and dorsal portions of the spinal cord.

## Competing interests

The authors declare that they have no competing interests.

## Authors' contributions

MZ drafted the manuscript, performed step-back experiments on vibrating probe, analyzed time-dependent injury current and step-back data and performed biophysical calculations and derived the equations for instantaneous surface injury currents. PL-S performed time-dependent injury current experiments on the spinal cords. AuH trained and provided support on the vibrating probe as well as aided in the biophysical analysis of the data and experimental setup. MP has been involved in analysis and interpretation of the data, as well as contributing to the drafting and revising of the manuscript. RBB is the Principle Investigator and Director of the CPR and is responsible for all elements of the research, as well as drafting and revising the final manuscript. All authors read and approved final manuscript.
